# Validation of mid-arm circumference for surveillance of obesity in African adolescent girls and adult women

**DOI:** 10.1017/S0007114523000387

**Published:** 2023-10-28

**Authors:** Hilde L. Nashandi, Makama A. Monyeki, John J. Reilly

**Affiliations:** 1 Physical Activity, Sport and Recreation Research Focus Area, Faculty of Health Sciences, North-West University, Potchefstroom 2520, South Africa; 2 School of Nursing and Public Health, Faculty of Health Sciences and Veterinary Medicines, University of Namibia, Windhoek, Namibia; 3 JJ Reilly, School of Psychological Sciences and Health, University of Strathclyde, Glasgow, Scotland

**Keywords:** Obesity, Body composition, Deuterium dilution method, Adolescent girls, Adult women, Anthropometry, Mid-upper arm circumference

## Abstract

This study aimed to assess the validity of mid-arm circumference (MAC), also known as mid-upper arm circumference (MUAC), for classification of high body fatness in Namibian adolescent girls and women and to test whether classification accuracy of MUAC was higher than the traditional simple proxy for high fatness, the BMI. In 206 adolescent girls aged 13–19 years and 207 adult women aged 20–40 years, we defined obesity conventionally (BMI-for-age Z score ≥ 2·00, adolescents; adults BMI ≥ 30·0 kg/m^2^) and also defined obesity using published MAC cut-off values. ^2^H oxide dilution was used to measure total body water (TBW) to define high body fat percentage (≥ 30 % in the adolescents, ≥ 38 % in the adults), and we compared the ability of BMI and MAC to classify high body fatness correctly using sensitivity, specificity and predictive values. In the adolescents, obesity prevalence was 9·2 % (19/206) using BMI-for-age and 63·2 % (131/206) using TBW; sensitivity of BMI-for-age was 14·5 % (95 % CI 9·1, 22·0 %) but was improved significantly using MAC of 22·6 cm (sensitivity 96·9 %; 95 % CI 92·1 %, 99·3 %). In the adults, obesity prevalence was 30·4 % (63/207) using BMI and 57·0 % (118/207) using TBW, and sensitivity of BMI was 52·5 % (95 % CI 43·6, 62·2 %), but using a MAC of 30·6 cm sensitivity was 72·8 % (95 % CI 66·4, 82·6 %). Surveillance of obesity in African adolescent girls and adult women is likely to be improved substantially by use of MAC as an alternative to the BMI-for-age and BMI.

Many African countries have seen large increases in the prevalence of obesity in the past few decades, even in children and adolescents and in rural areas^(1–3)^. Underweight remains common in many African countries, but in Namibia, for example, the prevalence of BMI-defined overweight and obesity in adult women already greatly exceeded that of underweight by 2012^(4)^. A more recent study using BMI among employees in a large company in Namibia found a prevalence of overweight and obesity combined of 74 %^(2)^. Across Africa, overweight and obesity prevalence is much higher in girls and women than in boys and men^(3,5–8)^. High prevalence of obesity, and particularly high risk in girls and women, will make efforts to prevent and control non-communicable diseases (including diabetes, heart disease, stroke, dementias, many cancers) more challenging for both the current generation and future generations^(9)^. Obesity is fuelling the African non-communicable disease crisis, and this is a current problem as well as a future concern: the leading causes of death among women in Namibia by 2017 were cerebrovascular diseases, ischaemic heart diseases, diabetes and hypertension^(10)^.

Surveillance of obesity prevalence is vital to obesity prevention and control^(9)^. For example, surveillance is essential to understand the causes of obesity, to identify trends and inequities, to develop policy and assess the impact of changes in policy and to appraise the impact of phenomena such as the COVID-19 pandemic. Measurement of BMI is the cornerstone of obesity surveillance nationally and globally, but obesity is high body fatness and BMI is only a proxy for high body fatness. Systematic reviews in children and adolescents^(11–14)^ have shown that BMI-for-age and waist circumference for age typically have high specificity (low false positive rates) but suffer from low-moderate sensitivity when used to classify high body fatness. Many children, adolescents and adults with apparently ‘healthy’ BMI-for-age, BMI, waist circumference for age or waist circumference actually have extremely high body fatness, sufficient to markedly increase their risk of cardiometabolic disease^(11–14)^, and this low-moderate sensitivity problem is increasingly being referred to as ‘normal weight obesity’ and creates the false impression that obesity (high body fatness) prevalence is much lower than it actually is. The extent to which the low-moderate sensitivity of BMI distorts estimates of obesity prevalence appears to vary between different populations, though studies from non-European populations are scarce^(15–18)^. In our recent study of children across Africa, the prevalence of high body fatness (measured accurately from ^2^H oxide measures of total body water (TBW)) was three times higher than the prevalence of obesity as defined by BMI-for age^(18)^ so low sensitivity of high BMI-for-age may be more of a problem in African populations than in the European populations which are over-represented in the literature^(11–15)^.

Mid-arm circumference (MAC), also known as mid-upper arm circumference (MUAC), is a well-established simple measure of undernutrition in low- and middle-income countries. The MAC has emerged recently as a promising proxy for high body fatness in public health surveillance^(19–22)^ but has not yet been validated against a reference measure of body fatness in any population. In children, adolescents and adults, the only reference method for measuring body fatness which is practical for use outside the laboratory is the measurement of TBW from ^2^H oxide dilution^(16,18,23)^. The present study therefore aimed to validate MAC against TBW-measured body fatness for classification of high body fatness and to assess whether MAC performs more adequately than the BMI-for-age/BMI for classification of obesity (high body fatness) in adolescent girls and adult women in Namibia.

## Methods

### Sample, study participants and ethics

The study protocol was reviewed and approved by the Namibian Ministry of Health and Social Services (MoHSS) Ethical Research Committee (ethical number: 17/3/3/ MV). Adult participants signed consent forms to participate in the study, while adolescents’ parents signed consent forms on their behalf and the adolescents provided verbal assent and this was recorded formally. Participants were informed that they were free to withdraw from the study at any time without consequence. All collected data were treated with strict confidentiality and study participants were informed that access to the data would be limited to MoHSS staff, expertise helping the project and University of Namibia staff who were involved directly in the study.

Since obesity prevalence seems higher among urban populations in Namibia, the present study recruited from urban settings only. Adolescent participants were drawn from four urban public schools in Khomas region (representing medium-high socio-economic status environments) using stratified random sampling of schools. From participating schools, adolescent girls were randomly selected and invited to take part: 207 adolescent girls participated chosen randomly from school registers and in age group 13–19 years. Only those girls whom their parents signed consent were recruited to the study. For the adults, 212 young women aged 20–40 years participated in the study and they were chosen randomly from teachers and staff members – using staff lists – of the 18 urban public schools in Khomas region.

All activities and procedures for the study such as interviews using questionnaires, anthropometric measurements and saliva samples (for the ^2^H dilution technique) took place at schools according to the agreed-upon project time schedule with all the participants.

### Weight status assessed using simple proxy measures in adolescents and adults

In the adolescents and adults, two trained researchers measured height to 0·1 cm and weight (in light indoor clothing) to 0·1 kg using standard methods and standard operating procedures^(18)^. An Infant/Child/Adult Portable Height-Length Measuring Board was used to measure height. Weight was measured using an electronic SECA scale, and the participants were asked to remove their footwear (shoes, slippers, sandals and socks; to have light clothes on and no items in the pockets). The BMI was calculated as kg/m^2^, and in the adults, obesity was defined as BMI > 30·0 kg/m^2^; in the adolescents, BMI-for-age Z score was calculated using WHO reference data and obesity was defined as BMI-for-age Z score ≥ 2·00, while overweight was defined as a BMI-for-age Z score of ≥ 1·00.

In the adolescents and adults, MAC was measured by a trained observer on the non-dominant arm^(20)^ with an adult plastic MAC tape which is non-stretchable and non-tear-resistant, at the midpoint between the olecranon and the acromial process after the arm is flexed to 90° from the elbow. Then, the arm was relaxed, the MAC tape was placed around the marked midpoint of the arm, neither too loose nor too tight, and the measurement was recorded to the nearest 0·1 cm. A MAC cut-off of 22·6 cm has been proposed for identification of excessive fatness in adolescent girls from South Africa^(20,24)^ and so this was used as a proxy definition of obesity in the present study. In adult women in Southern Africa, MAC cut-offs of 30·6 cm and 33·0 cm have been recommended to define obesity in women presenting for obstetric care at booking visits in early pregnancy^(24–27)^ so both were also used to define obesity in the adults in the present study.

Waist circumference was measured to 0·1 cm in the adults and adolescents using Lufkin measurement tape at the mid-point between iliac crest and lowest rib. Various cut-offs to define obesity based on waist circumference among adult women have been suggested, and in the present study, we used the lower and higher cut-offs from the WHO and International Diabetes Federation − 80·0 and 88·0 cm^(28,29)^ − as proxies for obesity, and also a higher cut-off which was derived from a study of associations between waist circumference and cardiometabolic risk factors in South African adult women of 91·5 cm^(30,31)^. Validated consensus cut-offs for high waist circumference among non-European adolescents are not available at present, but a waist circumference cut-off of 80·0 cm has been suggested for European adolescent girls^(29)^ and so that was tested among the adolescents in the present study. Since the validation of waist circumference was a secondary aim of the present study, results will be presented as online supplementary material. All anthropometric measurements were made in duplicate, and the mean value used in calculations.

### Body fatness measurement in adolescent and adults

In both the adolescents and adults, TBW was measured to 0·1 kg by measurement of ^2^H oxide dilution space. Full details of the measurement of TBW by ^2^H oxide dilution are provided elsewhere^(18,32)^. In brief, the TBW measures were made on the same day as the height, weight and waist circumference measures. An accurately weighed dose (to 0·0001 g, Ohaus Explorer Scale Pro EP214) of 0·5 g of ^2^H oxide labelled water (99·8 % purity, Cambridge Isotope Laboratories Inc.) per kg body weight was orally administered according to their body weight followed by 100 ml of local tap water. Adolescents and adults were asked not to eat or drink for at least 30 min before receiving the ^2^H-labelled water and to void their bladders prior to dosing. Baseline (pre-dose) saliva samples were collected from each participant by rotating a cotton wool ball in the buccal cavity of the mouth until well soaked and saliva collected into a clean sterile and dry tube using a 20 ml disposable syringe. Participants were then requested to drink the labelled water dose under supervision, and two further saliva samples (collected using the same method as described above) were collected at +3 h and +4 h after the dose. All saliva samples were stored at 4°C until return to the lab for storage at –20°C until analysis. Analysis was carried out with Fourier transform IR spectroscopy in accordance with International Atomic Energy Agency (IAEA) protocols^(18,32,33)^.

Since adolescents are still growing, the hydration of fat-free mass is declining^(32,33)^, and so TBW-derived measures of body fat mass were obtained by applying standard age and sex-specific constants for the hydration of fat-free mass as described previously^(17)^. In the adults, once growth has stopped, a constant for hydration of fat-free mass is appropriate and so we used the standard value for hydration of fat-free mass of 73·2 %^(32)^ to measure fat mass from TBW.

### Statistical analyses

Statistical Package for Social Sciences (SPSS® V25) was used to analyse the data. The sensitivity and specificity of BMI, waist circumference and MUAC against TBW measures of high body fatness were calculated. For adolescents, the cut-off of ≥ 30 % body fat was the body fatness measure of obesity as this has been used widely^(11–13,18)^ and is associated with marked increases in cardiometabolic risk factors^(3,4)^. Body fatness typically increases with age, and in the adults, a cut-off of ≥ 38 % body fat was used to define high body fatness – this cut-off has also been used widely and is associated with significantly elevated risk for cardiometabolic diseases in African-American women^(35)^.

We calculated the standard classification accuracy statistics as described by Akobeng *et al.*^(36)^: sensitivity, the % or proportion of people with disease who will have a positive result for the disease using a given index test; specificity, the % or proportion of people without the disease who will have a negative result; positive predictive value, the % or proportion of people with a positive test result who actually have the disease; negative predictive value, the % or proportion of people with a negative test result who do not have disease.

Power of the study was difficult to assess at the onset and was determined largely by practical considerations, but we note that the sample size for both the adolescents and adults was larger than in many previous studies of classification accuracy summarised in systematic reviews^(11–14)^ and that misclassification (underestimation) of high body fatness by BMI-for-age is often substantial^(18)^, suggesting that it might be identifiable with relatively small sample sizes. We also provide confidence intervals for sensitivity estimates so that the statistical significance of differences in sensitivity between the proxy measures can be interpreted.

## Results

### Characteristics of study participants

Characteristics of study participants are summarised in Table 1. A total of 207 adolescents and 212 adults took part in the study, and 206 adolescents and 207 adults actually provided complete data.

**Table 1.** Characteristics of study participants (mean values and standard deviations)

Variable	Adolescents (*n* 206)		Adults (*n* 207)	
	Mean	sd	Mean	sd
Age (years)	16·5	1·6	31·5	5·8
Height (cm)	159·9	5·9	161·7	5·9
Weight (kg)	56·9	13·6	71·2	17·0
BMI (kg/m^2^)	22·2	4·9	27·1	6·3
BMI Z-score	0·22	1·28	Not applicable	
MAC (cm)	26·2	4·0	30·5	4·6
Waist circumference (cm)	72·9	11·0	87·1	14·4
TBW (kg)	27·7	4·0	31·0	4·6
Fat mass (kg)	19·8	9·5	28·8	12·0
Fat-free mass (kg)	37·1	5·5	42·4	6·2
Body fat (% of body weight) from TBW	33·4	7·9	34·0	7·7

MAC, mid-arm circumference; TBW, total body water.

### Prevalence of obesity based on body fatness and proxies for body fatness

#### Adolescents

Prevalence of obesity based on BMI-for-age (BMI Z-score ≥ 2·00) among the adolescents was 9·2 % (*n* 19/206). Prevalence of high body fatness in the adolescents was 63·6 % (*n* 131/206). Prevalence of obesity according to MAC in the adolescents was 83·0 % (*n* 171/206).

#### Adults

In the adults, the prevalence of obesity defined as BMI ≥ 30·0 kg/m^2^ was 30·4 % (*n* 63/207). Prevalence of high body fatness was 57·0 % (*n* 118/207) when using the body fatness measure and cut-off ≥ 38 %, while prevalence of obesity at MAC of 33·0 cm was 31·4 % (*n* 65/207) and 44·0 % (*n* 91/207) at a MAC of 30·6 cm.

### Classification accuracy of simple anthropometric proxies for obesity

Classification accuracy data are summarised in Table 2 (adolescents) and Table 3 (adults).

**Table 2.** Adolescents: classification accuracy of body fatness percentage, BMI-for-age, MAC (Numbers and percentages)

Variable	Yes		No		Total
	*n*	%	*n*	%	
High body fatness (≥ 30·0 %)	131	63·6	75	36·4	206
BMI Z score ≥ +2·0	19	9·2	187	90·8	206
High MAC (≥ 22·6 cm)	171	83·0	35	17·0	206

MAC, mid-arm circumference.

**Table 3.** Adults: classification accuracy of body fatness percentage, BMI, MAC and waist circumference (Numbers and percentages)

Variable	Yes		No		Total
	*n*	%	*n*	%	
High body fatness (≥ 38·0 %)	118	57	89	43	207
BMI ≥ 30·0 kg/m^2^	63	30·4	144	69·6	207
High MAC (≥ 33·0 cm)	67	32·4	140	67·6	207
High MAC (≥ 30·6 cm)	91	44·0	116	56	207

MAC, mid-arm circumference.

#### Adolescents: BMI-for-age

In the adolescents, BMI-for-age Z-score of ≥ 2·00 had a sensitivity of 14·5 % (95 % CI 9·1, 22·0 %; of the 131 individuals with high body fatness, only 19 had a BMI-for-age which would define them as having obesity) and specificity of 100 % (of the seventy-five individuals who did not have high body fatness, all seventy-five had a BMI Z score which would not define them as having obesity). Positive predictive value for BMI-for-age defined obesity was 100 % (of the 19 with positive tests for obesity, 19 had high body fatness), while negative predictive value for BMI-for-age defined obesity was 39·6 % (of the 187 individuals who tested negative for obesity according to BMI-for-age, seventy-four did not have high body fatness).

#### Adolescents: mid-arm circumference

With MAC, the sensitivity for defining high body fatness among the adolescents was 96·9 % (95 % CI 92·0, 99·1 %; of the 131 individuals with high body fatness, 127 had MAC above the cut-off used), specificity was 46·7 % (of the seventy-five individuals who did not have high body fatness, thirty-five had a MAC below the cut-off < 22·6 cm). Positive predictive value for high MAC ≥ 22·6 cm defined as obesity was 72·3 % (of the 171 with positive tests for high MAC, 127 had high body fatness), while negative predictive value for individuals with MAC below cut-off was 88·6 % (of the thirty-five individuals who tested negative for obesity according to low MAC, thirty-one did not have high body fatness).

#### Adults: BMI

In the adults, the sensitivity and specificity of BMI ≥ 30·0 kg/m^2^ were 52·5 % (95 % CI 43·6, 62·2 %; 62/118 individuals with high body fatness had BMI ≥ 30·0 kg/m^2^) and 98·8 %, respectively (88/89 individuals who did not have high body fatness had a BMI < 30·0 kg/m^2^). Positive predictive value for BMI ≥ 30·0 kg/m^2^ defined obesity was 96·8 % (of the 63 with positive tests for obesity, 61 had high body fatness), while negative predictive value for BMI < 30·0 kg/m^2^ was 60·4 % (of the 144 individuals who tested negative for obesity according to BMI, eighty-seven did not have high body fatness).

#### Adults: mid-arm circumference

Sensitivity and specificity of a MAC cut-off of ≥ 30·6 cm in adults were 72·8 % (95 % CI 66·4, 82·6 %; 86/118) and 94·4 % (84/89) respectively, while for a MAC cut-off of 33·0 cm, the sensitivity and specificity were 56·8 % (95 % CI 47·3, 65·8 %; 67/118) and 95·5 % (85/89), respectively. Positive predictive value for high MAC ≥ 30·6 cm was 93·4 % (of the ninety-one with positive tests for high MAC, eighty-five had high body fatness), while negative predictive value for individuals with MAC below this cut-off was 68·9 % (of the 116 individuals who tested negative for obesity according to low MAC, eighty did not have high body fatness).

Positive predictive value for MAC ≥ 33·0 cm was 100·0 % (of the sixty-seven with positive tests for high MAC, sixty-seven had high body fatness), while negative predictive value for individuals with MAC below cut-off was 62·1 % (of the 140 individuals who tested negative for obesity according to low MAC, eighty-seven did not have high body fatness).

## Discussion

### Main findings and study implications

The present study with adolescent girls and adult women suggests that obesity prevalence estimation by BMI and BMI-for-age in African populations is highly conservative and should be used with caution^(15)^. The degree of misclassification when using BMI and BMI-for-age in the present study was substantial, and this will be problematic for other applications beyond surveillance, for example, if BMI and BMI-for-age are used as exposure or outcome variables in epidemiological studies.

The major advance in the present study is the evidence that, with validation against a reference method of measuring body fatness (TBW as measured by ^2^H oxide dilution), MAC outperformed BMI and BMI-for-age for classification of high body fatness in both the adolescents and the adults. The present study suggests that MAC does not suffer from the same problem of low sensitivity that BMI/BMI-for-age suffers from when used to classify high body fatness in African populations. The major surveillance problem with BMI and BMI-for-age is low sensitivity – for surveillance purposes (prevalence estimates) the sensitivity of the simple anthropometric proxy will be more important than specificity. The degree of misclassification when using MAC varied by cut-off chosen, and in the present study, we restricted ourselves to testing the classification accuracy of published MAC cut-offs only. Alternative cut-offs may provide even higher classification accuracy, but these will need to be developed by future larger primary studies or data-pooling studies.

### Comparisons with other studies

Systematic reviews of the classification accuracy of simple epidemiological definitions of obesity in children and adults^(11–14)^ have found that very few previous studies used accurate (reference) methods of body composition to estimate classification accuracy, few considered multiple simple proxies (BMI, MAC, waist circumference) in the same study and almost no studies were conducted in African populations, a particular concern given the likely population-specific influences on classification accuracy^(14,16,18)^. A recent systematic review of the ability of MUAC to classify overweight and obesity in children and adolescents^(22)^ found many studies of the ability of MUAC to classify individuals having obesity based on BMI-for-age, but a dearth of evidence (no eligible studies) on the ability of MUAC to classify body fatness-defined obesity using a reference method and called for more evidence of the kind provided by the present study. The present study is consistent with our previous study with children across Africa^(18)^ and with a large body of evidence from mainly European populations suggesting that use of BMI (in adults) and BMI-for-age (in children and adolescents) is highly conservative when estimating prevalence of obesity, that is, high body fatness^(11–14)^.

### Strengths and weaknesses of the present study

The present study had a number of strengths and weaknesses. The main strengths were the novelty of the African sample, the unique use of a reference method for measurement of body fatness against which the simple proxies could be validated – previous classification accuracy studies have tested the accuracy of a method of unknown or low validity against another method of low or unknown validity as noted above^(18,22)^. Our ability to test the classification accuracy of a wide range of simple anthropometric proxies (previously published, used widely and/or recommended) within the same sample was also a notable strength.

Of the study weaknesses, generalisability of our findings is unclear. For example, we are unable to comment on generalisability to adolescent boys and adult men in Namibia, but the much lower prevalence of obesity among boys and men compared with girls and women in Sub-Saharan Africa makes this issue less urgent. Generalisability to other populations from different geographical regions (e.g. Asia, Europe/ North America) is also uncertain, but the consistency of our findings with systematic reviews of the modest classification accuracy of BMI and BMI-for age^(11–14)^ in other human populations suggests that there may be a high degree of generalisability. The recruitment of an urban sample in the present study was partly based on convenience, and partly to ensure that study participants had a wide range of body fatness, including many participants with high body fatness. Recruitment from rural areas would have been less practical and would have produced far fewer participants with high body fatness, so limiting our ability to test the classification accuracy of MAC.

Future, larger, studies of both sexes will be required to identify the optimum cut-offs in the BMI/BMI-for-age, MAC and waist circumference distribution to classify high body fatness and to examine the extent to which misclassification varies systematically between different human populations. It is possible that optimising the classification accuracy of MAC in children and adolescents will require age-specific cut-offs. In the previous studies which have assessed the classification accuracy of MAC for defining overweight and/or obesity as defined by the BMI-for-age in children and adolescents, the classification accuracy and optimal MAC cut-offs varied to some degree by age^(19,37,38)^. The present study aimed to test the classification accuracy of nine published and/or currently recommended cut-offs (three in the adolescents using BMI-for-age, MAC and waist circumference; six in the adults, one based on BMI, two based on MUAC, three based on waist circumference), rather than propose new cut-offs. We considered that the development of new MAC cut-offs using receiver operator curve analysis – including the development of age-specific cut-offs – was beyond the scope of the present study, and study power for the development of new MAC cut-offs quite limited.

Since the present study had a focus on testing the ability of MAC and waist circumference for improving the problem of low sensitivity of BMI and BMI-for-age for estimating obesity prevalence, our emphasis was on sensitivity of classification. For other purposes (e.g. classification of individual as having or not having obesity in clinical settings) other aspects of classification accuracy will be more important, and it should be noted that specificity of BMI and BMI-for-age was high, as expected^(11–14,18)^, suggesting low rates of misclassification if used for clinical purposes.

While we provided prevalence estimates in the present study, the focus was not on those estimates *per se*; but to demonstrate the possible effect on prevalence estimates of using BMI *v*. MAC to define obesity, the effect is likely to be large. Whether or not MAC is adopted for surveillance of obesity (e.g. in national or international surveys or surveillance studies) will depend on factors other than classification accuracy. Practical issues such as cost/availability and measurement accuracy in the field will also be important. Since MAC is already so practical, well established and widely used across Africa to assess undernutrition, it should be a promising option for obesity surveillance on practical grounds too. The issue of why BMI/BMI-for-age performs so poorly in classification of high body fatness, and why this is an even greater problem in some populations than others, was beyond the scope of the present study. Evidence on between-population variation in the hydration of fat-free mass is scarce at present, but while there appear to be small systematic variations in fat-free mass hydration by age, sex and weight status, systematic population-specific variations appear to be very small or non-existent^(39,40)^. We used the IAEA online handbook for age-specific and sex-specific hydration constants for fat-free mass^(33)^, which incorporates the most recent and comprehensive data available on the composition of fat-free mass in order to convert measures of TBW to measures of total body fat^(40)^.

### Conclusions

In summary, the present study suggests that MAC can offer substantially improved obesity surveillance compared with the standard approach based on BMI in Africa. The conservative nature of defining obesity based on BMI-for-age and BMI is underestimating the scale of the obesity problem in Africa, and this underestimation may be hindering or distorting public health responses to the obesity pandemic.

## Supplementary material

For supplementary material accompanying this paper visit https://doi.org/10.1017/S0007114523000387. click here to view supplementary material
