# Supplementary material for: Validation of mid-arm circumference for surveillance of obesity in African adolescent girls and adult women
Source: Br J Nutr. 2023 Mar 9;130(8):1437–43. doi: 10.1017/S0007114523000387 (PMC10511682; doi:10.1017/S0007114523000387)
Supplement: Supplementary file 1 [file S0007114523000387sup001.docx]

**Supplementary File 1 Waist Circumference Data**

**Prevalence data, adolescents**. The prevalence of obesity according to waist circumference (>80.0cm cut-off) was 20.3% (n= 42/206).

**Prevalence data, adults**. Prevalence of obesity according to waist circumference among the adults was 63.8 % (n=132/207), 46.4% (n= 96/207) and 39.1 % (n= 81/207) at waist circumference cut-offs of 80.0cm, 88.0cm and 91.5cm respectively. .

**Classification accuracy data**

**Adolescents-Waist Circumference:** Among adolescents, the sensitivity of waist circumference >80.0cm was 32.6% (CI 8.2%; of the 131 individuals with high body fatness, 42 had a WC of >80.0cm) but specificity was 56.0 % (of the 75 individuals who did not have high body fatness, 42 have a WC below the cut-off < 80.0cm).

Positive predictive value for high WC >80.0cm defined as obesity was 100.0% (of the 42– with positive tests for high WC, all 42 had high body fatness), while Negative predictive value for individuals with WC below cut-off was 20.1% (of the 164 individuals who tested negative for obesity according to low WC <80.0cm, 33 did not have high body fatness).

**Adults-Waist Circumference:** Sensitivity and specificity with a waist circumference higher cut off of 91.5cm was 67.8 % (CI 8.4%; 80/118) and 97.7 % (87/89) respectively. Positive predictive value for high waist circumference ≥91.5cm defined as obesity was-97.5 % (of the 81 individuals with positive tests for high waist circumference, 79 had high body fatness), while Negative predictive value for individuals with waist circumference below cut-off was 69.8 % (of the 126 individuals who tested negative for obesity according to low waist circumference, 88 did not have high body fatness).

The sensitivity and specificity of a waist circumference with cut off of ≥88.0cm was 74.6 % (CI 7.9%; 88/118) and 87.6 % (78/89) respectively. Positive predictive value for high waist circumference ≥88.0cm defined as obesity was 94.8% (of the 96 individuals with positive tests for high waist circumference, 94 had high body fatness), while Negative predictive value for individuals with waist circumference below cut off of <88.0cm was 71.2 % (of the 111 individuals who tested negative for obesity according to low waist circumference, 79 did not have high body fatness).

The sensitivity and specificity of a waist circumference with cut off of ≥80.0cm was 90.7 % (CI 5.2%; 107/118) and 68.5 % (61/89) respectively. Positive predictive value for high waist circumference ≥80.0cm defined as obesity was 79.5 % (of the 132 individuals with positive tests for high waist circumference, 105 had high body fatness), while Negative predictive value for individuals with waist circumference below cut-off was 82.7 % (of the 75 individuals who tested negative for obesity according to low waist circumference, 62 did not have high body fatness).
